# Assessment of ISO Method 15216 to Quantify Hepatitis E Virus in Bottled Water

**DOI:** 10.3390/microorganisms8050730

**Published:** 2020-05-13

**Authors:** Enric Cuevas-Ferrando, Antonio Martínez-Murcia, Alba Pérez-Cataluña, Gloria Sánchez, Walter Randazzo

**Affiliations:** 1 Department of Preservation and Food Safety Technologies, IATA-CSIC, Av. Agustín Escardino 7, 46980 Paterna, Valencia, Spain; enric.cuevas@iata.csic.es (E.C.-F.); alba.perez@iata.csic.es (A.P.-C.); gloriasanchez@iata.csic.es (G.S.); 2 Area de Microbiología, EPSO, Universidad Miguel Hernández, Carretera de Beniel Km 3.2, 03312 Orihuela, Alicante, Spain; ammurcia@umh.es; 3 Department of Microbiology and Ecology, University of Valencia. Av. Dr. Moliner, 50, 46100 Burjassot, Valencia, Spain

**Keywords:** Hepatitis E Virus (HEV), bottled water, concentration method, RT-qPCR

## Abstract

Hepatitis E virus (HEV) is one of the causative agents of water-borne human viral hepatitis and considered in Europe an emerging zoonotic pathogen. Analysis of bottled water through a standard method validated for HEV can contribute towards the risk management of this hazard. Putting some recent reports by the European Food Safety Authority in place, this study aimed to assess the performance of the concentration and extraction procedures described in ISO 15216-1:2017 for norovirus and hepatitis A virus on HEV detection. Following the ISO recommendation, the bottled water samples were spiked using serially diluted HEV fecal suspensions together with mengovirus as process control and concentrated by filtration via positively charged nylon membranes. In order to extract viral RNA from the resulting concentrates, two different methods were compared in this study: The one recommended in the ISO norm, NucliSens^®^ MiniMag^®^ system (NS), and an alternative commercially available kit NucleoSpin^®^RNA virus kit (MN). Finally, three reverse transcription quantitative PCR (RT-qPCR) assays were used to quantify HEV titers. The evaluated procedures resulted in average HEV recoveries of 14.08 ± 4.90% and 3.58 ± 0.30% for the MN and NS methods, respectively. The limit of detection (LoD_95%_) was 1.25 × 10^4^ IU/L for both extraction methods combined with the three RT-qPCR assays tested, with the exception of NS extraction coupled with RT-qPCR1 that showed a LoD_95%_ of 4.26 × 10^3^ IU/L. The method characteristics generated in this study support the limited suitability of the ISO 15216-1:2017 concentration procedure coupled with the evaluated RT-qPCR assays for detecting HEV in bottled water.

## 1. Introduction

Hepatitis E virus (HEV) is either a quasi- or non-enveloped, single-stranded, positive-sense RNA virus that causes acute hepatitis [1]. Globally, it has been estimated that HEV causes 3 million acute cases and 56,600 HEV-related deaths per year [2]. In Europe, hepatitis E is considered an emerging infectious disease given the increasing trend of outbreaks in the past decade [3].

HEV causes water- and food-borne diseases with different epidemiology depending on the geographical region and the circulating genotype. HEV genotype G1 and G2 are responsible for most of hepatitis E cases in highly endemic areas, causing large outbreaks mostly associated with the consumption of contaminated water. By contrast, HEV G3 and G4 causes sporadic cases or small outbreaks in non-endemic areas, corresponding to low endemic countries [4,5,6]. Sporadic and epidemic HEV outbreaks in endemic regions have been mostly caused by waterborne transmission, while the zoonotic transmission has been demonstrated in developed countries mainly due to the consumption of raw pork and wild-boar meat [5,7,8].

HEV waterborne outbreaks have occurred in poor sanitation and hygiene practices settings where the access to drinking water is limited [9,10,11]. HEV could be transported along all the water cycle by runoff, drainage, and overland flow, and potentially contaminate surface waters used for drinking water production [12]. Even though some experimental information supports the effectiveness of UV and chlorine disinfection treatments against HEV based on the partial reduction of the RNA copies [13,14], the lack of standardized procedures for HEV detection in bottled water hampers the development of risk management tools and thus the policy on water safety. Moreover, by applying a standardized and official method, researchers could generate more useful harmonized data.

Concerning this matter, the European Food Safety Authority (EFSA) recently stated that methods described in the validated ISO 15216-1:2017 norm to quantify hepatitis A virus (HAV) and human norovirus genogroup I and II (norovirus GI and GII) [15,16] should be evaluated to demonstrate their effectiveness for the detection of HEV in bottled water and food matrices [3]. The same agency recently quoted foodborne viruses and specifically HEV among the topics of the food safety regulatory research needs 2030 [17]. Therefore, this study aimed to assess the ISO 15216-1:2017 method for detecting HEV in bottled water as well as some modifications of the procedure and to generate method characteristics such as the virus recovery efficiencies and the limit of detection.

## 2. Materials and Methods

### 2.1. Virus Strains

Human fecal sample containing HEV genotype 3f was used in this study. Fecal sample (10% *w*/*v*) was suspended in phosphate-buffered saline (PBS) containing 2 M NaNO_3_ (Panreac, Barcelona, Spain), 1% beef extract (Conda, Madrid, Spain), and 0.1% Triton X-100 (Thermo Fisher Scientific, Barcelona, Spain) (pH 7.2). The mix was then vigorously vortexed and centrifuged at 1000× *g* for 5 min to obtain a final 10% (*w*/*v*) fecal suspension in PBS. The supernatant was stored at −80 °C in 2 mL aliquots. The first WHO international standard for HEV nucleic acid amplification technique (NAT)-based assays (code 6329/10) was purchased from Paul-Ehrlich-Institut (Langen, Germany). This standard corresponds to HEV genotype 3a positive plasma measured in international units and containing 250,000 international units (IU)/mL [18]. Mengovirus (MgV) vMC_0_ (CECT 100000, Valencia, Spain) was used as a process control. 

### 2.2. Detection Limit and Efficiency of the Procedure to Concentrate HEV in Bottled Water according to ISO 15216-1:2017

Commercial bottled water samples were artificially inoculated with serially diluted (5, 4, and 3 log_10_ IU/L) 10% HEV positive fecal suspension. To determine the efficacy of the procedures, and thus validate the results, bottled water samples were also spiked with titrated MgV (10^7^ PCRU/L) as process control [15,19]. Two experimental replicates were analyzed for each contamination level. Samples were then concentrated and processed as described by ISO 15216-1:2017. Briefly, 1 L of bottled water was inoculated with HEV and filtered through nylon positively charged membrane filter discs with 0.45 µm pore size and 47 mm diameter (3M Company, Spain) using a vacuum pump. 4 mL Tris-glycine-beef extract (TGBE) buffer pH 9.5 was used to elute the virus from the filter, 10 mL TGBE to elute the virus attached to the bottle by shaking 20 min at 50 rpm and 2 mL TGBE to rinse the interior walls of the bottle by gentle shaking and inversion by hand. All the eluates were pooled, pH adjusted to 7.0 with HCl and viruses concentrated by ultrafiltration with 30 kDa Amicon^®^ Ultra-15 filter tubes (Merck Millipore Ltd., Madrid, Spain). Final concentrates were adjusted to 1 mL with PBS and stored at −80 °C until processed. RNA was extracted with either the NucleoSpin^®^RNA (Macherey-Nagel GmbH & Co., Dueren, Germany) (referred to as MN) or the Nuclisens^®^ MiniMag^®^ (BioMérieux, Barcelona, Spain) (referred to as NS) kits according to the manufacturer’s instructions as detailed in Section 2.3. For each method and contamination level, a sample with PBS (i.e., without bottled water) was included in order to assess potential matrix effects.

### 2.3. RNA Extraction and RT-qPCR Assays

The MN extraction was performed according to the manufacturer’s instructions with some modifications. Briefly, 150 µL of each concentrated sample was mixed with 25 μL Plant RNA Isolation Aid (Thermo Fisher Scientific, Barcelona, Spain) and 600 μL of lysis buffer from the MN kit and subjected to pulse-vortexing for 1 min. Afterwards, the homogenate was centrifuged for 5 min at 10,000× *g* to remove the debris. The supernatant was subsequently processed according to the MN manufacturer’s instructions. The NS extraction was performed according to the manufacturer’s instructions; sample volume was 500 μL and elution volume was 100 μL.

Resultant RNA was analyzed using the RNA UltraSense One-Step kit (Invitrogen, Barcelona, Spain) and RT-qPCR performed as described by Schlosser [20] for HEV (referred as RT-qPCR1) and ISO 15216-1:2017 for MgV. For both assays, undiluted and 1/10 diluted RNA was tested to check for inhibitors. Moreover, RNAs were also quantified using the CeeramTools^®^ Hepatitis E Virus Detection KHEV commercial kit (BioMérieux) (referred as RT-qPCR2) provided with an internal amplification control and a HEV RT-qPCR assay described by Jothikumar [21] and modified by Girón-Callejas [22] (RT-qPCR3). All samples were run in duplicate and different controls were used, including negative process, extraction, and RT-qPCR controls. 

HEV was quantified by plotting the quantification cycles (Cqs) to an external standard curve independently built for each RT-qPCR assay with the International Standard WHO HEV RNA (code 6329/10). Relevant RT-qPCR1 and RT-qPCR2 assay characteristics have been reported elsewhere [23]. Standard curve for RT-qPCR3 (y = -3.5008x + 38.564) showed a R^2^ value of 0.997.

### 2.4. Statistical Analysis

Results were statistically analyzed, and significance of differences was determined on the ranks with a one-way analysis of variance (ANOVA) and Tukey’s multiple comparison tests. In all cases, a value of *p* < 0.05 was deemed significant. The estimated probability of detection with 95% confidence (LoD_95%_) was calculated by using the PODLOD calculation program (version9) [24] for all water samples, as in [16].

## 3. Results and Discussion

### Limit of Detection and Efficiency of HEV Concentration Procedure in Bottled Water Based on ISO 15216-1:2017

Given the aforementioned regulatory research priorities, the present study aimed to evaluate ISO 15216-1:2017 method for HEV detection in bottled water and to generate method characteristics as the virus recovery yield and the LoD_95%_.

To this end, inoculated bottled water samples were processed as described in ISO 15216-1:2017 and RNA was extracted using two commercially available kits, NucleoSpin^®^RNA virus kit and NucliSens^®^ MiniMag^®^ system. 

The resultant RNA was analyzed through three different RT-qPCR assays (Table 1): RT-qPCR1 (as described in [20,23], CeeramTools^®^ Hepatitis E Virus Detection HEV commercial kit (RT-qPCR2), and RT-qPCR3 as described in [21] and modified in [22].

**Figure 1:**
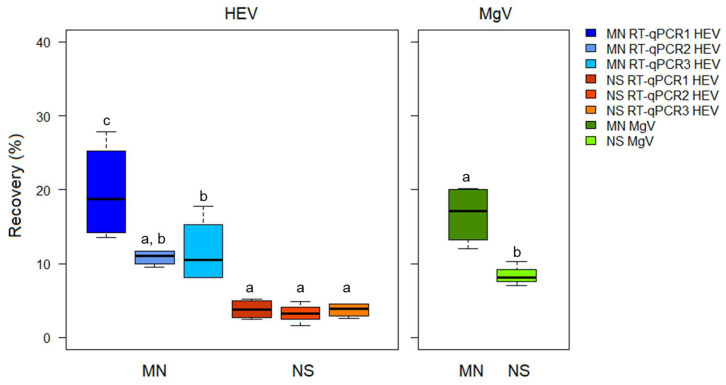
Hepatitis E virus (HEV) recovery in bottled water samples using the ISO 15216-1:2017 procedure and comparing two extraction kits and three RT-qPCRs assays. MN: NucleoSpin^®^RNA virus kit (Macherey-Nagel GmbH & Co.); NS: NucliSens^®^ MiniMag^®^ system (BioMerieux SA); RT-qPCR1: modified from [20]; RT-qPCR2: CeeramTools^®^ Hepatitis E Virus Detection KHEV kit (BioMérieux SA); and RT-qPCR3: Described in [21] and modified in [22]. Different letters denote significant differences among methods for each virus (*p* < 0.05).

The mean HEV recoveries obtained from the processed bottled water samples were 14.08 ± 4.90% and 3.58 ± 0.30% for the MN and NS methods, respectively (Figure 1). 

The MN method rendered a LoD_95%_ of 1.25 × 10^4^ IU/L for all the RT-qPCR assays, while the NS extraction coupled with RT-qPCR1 rendered a slightly lower LoD_95%_ of 4.26 × 10^3^ IU/L (Table 2). 

A minimum recovery rate of 1% MgV was obtained for all the samples, thus validating the results according to the criteria stated in ISO 15216-1:2017. Additional analytical determination should be performed by spiking purified HEV virus, since the fecal material used in the study is expected to contain defective particles and free RNA that may have negatively contributed to calculate the recoveries. Despite NS extraction showed lower HEV recoveries, it resulted more sensitive due to lower LoD_95%_.

Although HEV LoD_95%_ in bottled water are not available, those obtained in this study are somehow higher compared to the ones reported for HAV and norovirus in the method validation. For instance, LoD_95%_ of 1.80 × 10^2^, 7.00 × 10^1^, and 4.00 × 10^2^ copies/L have been reported for norovirus GI, norovirus GII, and HAV, respectively [16].

These discrepancies could be partially explained by the different dilution series used in the validation study (0.5 log_10_) [16] compared to those in the present study (1 log_10_). 

Furthermore, to compare the efficiencies of MN and NS extraction kits, we adjusted the final concentrated volume to 1 mL instead of 500 μL as indicated in the ISO 15216-1:2017. 

Despite reaching acceptable recovery values, this has certainly determined a decrease in the sensitivity of the assay by increasing the LoD_95%_. Comparing the LoD_95%_ values determined in this study for HEV in bottled water (approximately 10^3^‒10^4^ gc/L) with the ones in wastewater (approximately 10^5^‒10^6^ gc/L), effluent (approximately 10^4^ gc/L), and drinking water (approximately 10^3^ gc/L) [25], the effect of the complexity (i.e., turbidity, volume of the sample) of the water matrix results evident. 

Our results on the better performance of MN extraction resemble the previously reported recovery rates ranging from 16.6% to 36.6% obtained by using a dead end hollow fiber ultrafiltration (DEUF) combined with polyethylene glycol (PEG) precipitation to concentrate HEV spiked into 20 L drinking water [25]. 

Beside the overall performances of the concentration methods, these results confirm the higher sensitivity of RT-qPCR1 compared to RT-qPCR3, as previously reported for the detection of HEV in suspension containing the WHO international standard, in human fecal and serum samples, and in naturally contaminated sewage samples [23]. Similarly, RT-qPCR1 showed to be more sensitive in detecting HEV in spiked sewage samples, even RT-qPCR3 with a modified probe finally resulted the best option for wastewater monitoring in a one-year surveillance study [25].

Since RT-qPCR3 detects HEV genotypes one to six and RT-qPCR1 has been initially designed for porcine HEV-3, the efficiency of the RT-qPCR assays should be tested by mixing in drinking water different HEV genotypes. However, given the variability of HEV genotypes [26], the RT-qPCR assays used for naturally contaminated samples must be carefully checked to avoid false negative results and maybe primer-independent assays should be preferred (i.e., non targeted NGS techniques).

Previous results support DEUF coupled with PEG as an effective method for HEV concentration in large volume sample of drinking water [25] and it may represent a valuable alternative given the limited sensitivity and efficiency of the ISO method reported in this study. 

Finally, this study generated experimental data to characterize ISO method 15216-1:2017 performance when adapted to analyze HEV in bottled water, such as virus recovery efficiency and the limit of detection (LoD_95%_).

## 4. Conclusions

HEV is considered an emerging pathogen in high-income countries, especially in Europe, and analytical procedures for estimating HEV concentrations in water samples are required.

The method characteristics generated in this study support the limited suitability of the ISO 15216-1:2017 concentration procedure for recovering HEV in bottled water, even some modifications such as the reduction of the final concentrate volume could foster the overall analytic performance of the method.

## Figures and Tables

**Table 1:** Primers and probes used in this study.

Assay	Amplification Region	Primers and Probe	Sequence 5’-3’	RT-qPCR Conditions	Location *	Reference
RT-qPCR1	ORF3	HEV.Fa	GTGCCGGCGGTGGTTTC	RT 50 °C for 30’	5296–5377 (81 nt)	[20]
		Hev.Fb	GTGCCGGCGGTGGTTTCTG	95 °C for 15’		
		HEV.R	GCGAAGGGGTTGGTTGGATG	PCR (45x)		
		HEV.P	FAM-TGACMGGGT/ZEN/TGATTCTCAGCC/3IABkFQ	95 °C for 10’’		
				55 °C for 20’’		
				72 °C for 15’’		
RT-qPCR2	ORF3	N/A	N/A	RT 45 °C for 10’	N/A	Ceeram (hepatitis@ceeramTools)
				95 °C for 10’		
				PCR (40x)		
				95 °C for 15’’		
				60 °C for 45’’		
RT-qPCR3	ORF3	JVHEVF	GGTGGTTTCTGGGGTGAC	RT 50 °C for 30’	5304–5373 (69 nt)	[21,22]
		JVHEVRmod	AGGGGTTGGTTGGRTGRA	95 °C for 2’		
		JVHEVPmod	TGATTCTCAGCCCTTCGC	PCR (45x)		
				95 °C for 15’’		
				60 °C for 40’’		

F: Forward primer; R: Reverse primer; and P: Probe.* Location in reference to WHO International Standard for HEV RNA, HRC-HE104 strain, accession no. AB630970 [18].

**Table 2:** Limit of detection of HEV in bottled water according to ISO 15216-1:2017 virus concentration procedure and comparing two extraction kits and three RT-qPCRs assays.

Extraction Method	RT-qPCR	Levels of Inoculated HEV (IU/L)			LoD_95%_^a^ (IU/L)
		1 × 10^5^	1 × 10^4^	1 × 10^3^	
MN	RT-qPCR1	4/4 ^b^	4/4	0/4	1.25 × 10^4^
	RT-qPCR2	4/4	4/4	0/4	1.25 × 10^4^
	RT-qPCR3	4/4	4/4	0/4	1.25 × 10^4^
NS	RT-qPCR1	4/4	4/4	2/4	4.26 × 10^3^
	RT-qPCR2	4/4	4/4	0/4	1.25 × 10^4^
	RT-qPCR3	4/4	4/4	0/4	1.25 × 10^4^

^a^ Calculated according to (24); ^b^ HEV positive/total numbers of samples.

## References

[1] Wang Y., Zhao C., Qi Y., Geng Y. (2016). Hepatitis E virus. Advances in Experimental Medicine and Biology.

[2] World Health Orgnization (2014). Waterborne Outbreaks of Hepatitis E: Recognition, Investigation and Control.

[3] Ricci A., Allende A., Bolton D., Chemaly M., Davies R., Fernandez Escamez P.S., Herman L., Koutsoumanis K., Lindqvist R., Nørrung B. (2017). Public health risks associated with hepatitis E virus (HEV) as a food-borne pathogen. EFSA J..

[4] Fenaux H., Chassaing M., Berger S., Gantzer C., Bertrand I., Schvoerer E. (2019). Transmission of hepatitis E virus by water: An issue still pending in industrialized countries. Water Res..

[5] Lu L., Li C., Hagedorn C.H. (2006). Phylogenetic analysis of global hepatitis E virus sequences: Genetic diversity, subtypes and zoonosis. Rev. Med. Virol..

[6] Ruggeri F.M., Di Bartolo I., Ostanello F., Trevisani M. (2013). Hepatitis E Virus: An Emerging Zoonotic and Foodborne Pathogen.

[7] Van der Poel W. (2014). Food and environmental routes of Hepatitis E virus transmission. Curr. Opin. Virol..

[8] Van der Poel W., Rzezutka A., Hepatitis E., Meschke J.S., Girones R. (2019). Global Water Pathogen Project.

[9] Boccia D., Guthmann J.-P., Klovstad H., Hamid N., Tatay M., Ciglenecki I., Nizou J.-Y., Nicand E., Guerin P.J. (2006). High Mortality Associated with an Outbreak of Hepatitis E among Displaced Persons in Darfur, Sudan. Clin. Infect. Dis..

[10] Guerrero-Latorre L., Carratala A., Rodriguez-Manzano J., Calgua B., Hundesa A., Girones R. (2011). Occurrence of water-borne enteric viruses in two settlements based in Eastern Chad: Analysis of hepatitis E virus, hepatitis A virus and human adenovirus in water sources. J. Water Health.

[11] UNHCR UNHCR steps up measures to rein in Hepatitis E among South Sudanese refugees in Ethiopia. https://www.unhcr.org/news/.

[12] Givens C.E., Kolpin D.W., Borchardt M.A., Duris J.W., Moorman T.B., Spencer S.K. (2016). Detection of hepatitis E virus and other livestock-related pathogens in Iowa streams. Sci. Total Environ..

[13] Girones R., Carratalà A., Calgua B., Calvo M., Rodriguez-Manzano J., Emerson S. (2014). Chlorine inactivation of hepatitis e virus and human adenovirus 2 in water. J. Water Health.

[14] Guerrero-Latorre L., Gonzales-Gustavson E., Hundesa A., Sommer R., Rosina G. (2016). UV disinfection and flocculation-chlorination sachets to reduce hepatitis E virus in drinking water. Int. J. Hyg. Environ. Health.

[15] ISO 15216-1 (2017). ISO 15216-1:2017—Microbiology of the Food Chain—Horizontal Method for Determination of Hepatitis A Virus and Norovirus Using Real-Time RT-PCR—Part 1: Method for Quantification.

[16] Lowther J.A., Bosch A., Butot S., Ollivier J., Mäde D., Rutjes S.A., Hardouin G., Lombard B., In’t Veld P., Leclercq A. (2019). Validation of EN ISO method 15216—Part 1—Quantification of hepatitis A virus and norovirus in food matrices. Int. J. Food Microbiol..

[17] Bronzwaer S., Kass G., Robinson T., Tarazona J., Verhagen H., Verloo D., Vrbos D., Hugas M. (2019). Food Safety Regulatory Research Needs 2030. EFSA J..

[18] Baylis S.A., Sakata H., Okada Y., Mizusawa S., Hanschmann K.-M.O., Nübling C.M., Matsubayashi K., Blümel J., Mizusawa S., Matsubayashi K. (2013). World Health Organization International Standard to Harmonize Assays for Detection of Hepatitis E Virus RNA. Emerg. Infect. Dis..

[19] Gerba C.P., Betancourt W.Q., Kitajima M., Rock C.M. (2018). Reducing uncertainty in estimating virus reduction by advanced water treatment processes. Water Res..

[20] Schlosser J., Eiden M., Vina-Rodriguez A., Fast C., Dremsek P., Lange E., Ulrich R.G., Groschup M.H. (2014). Natural and experimental hepatitis E virus genotype 3-infection in European wild boar is transmissible to domestic pigs. Vet. Res..

[21] Jothikumar N., Cromeans T.L., Robertson B.H., Meng X.J., Hill V.R. (2006). A broadly reactive one-step real-time RT-PCR assay for rapid and sensitive detection of hepatitis E virus. J. Virol. Methods.

[22] Girón-Callejas A., Clark G., Irving W.L., McClure C.P. (2015). In silico and in vitro interrogation of a widely used HEV RT-qPCR assay for detection of the species Orthohepevirus A. J. Virol. Methods.

[23] Randazzo W., Vásquez-García A., Bracho M.A., Alcaraz M.J., Aznar R., Sánchez G. (2018). Hepatitis E virus in lettuce and water samples: A method-comparison study. Int. J. Food Microbiol..

[24] Wilrich C., Wilrich P.-T. (2009). Estimation of the POD function and the LOD of a qualitative microbiological measurement method. J. AOAC Int..

[25] Cuevas-Ferrando E., Randazzo W., Pérez-Cataluña A., Sánchez G. (2020). HEV Occurrence in Waste and Drinking Water Treatment Plants. Front. Microbiol..

[26] Smith D.B., Simmonds P., Izopet J., Oliveira-Filho E.F., Ulrich R.G., Johne R., Koenig M., Jameel S., Harrison T.J., Meng X.-J. (2016). Proposed reference sequences for hepatitis E virus subtypes. J. Gen. Virol..

